# Ion-Beam-Directed Self-Ordering of Ga Nanodroplets on GaAs Surfaces

**DOI:** 10.1186/s11671-016-1234-y

**Published:** 2016-01-27

**Authors:** Xingliang Xu, Jiang Wu, Xiaodong Wang, Mingliang Zhang, Juntao Li, Zhigui Shi, Handong Li, Zhihua Zhou, Haining Ji, Xiaobin Niu, Zhiming M. Wang

**Affiliations:** Institute of Fundamental and Frontier Science, State Key Laboratory of Electronic Thin Film and Integrated Devices, University of Electronic Science and Technology of China, Chengdu, 610054 People’s Republic of China; Institute of Electronic Engineering, China Academy of Engineering Physics, Mianyang, 621999 People’s Republic of China; Research Center for Microsystems and Terahertz, China Academy of Engineering Physics, Mianyang, 621999 People’s Republic of China; Engineering Research Center for Semiconductor Integrated Technology, Institute of Semiconductors, Chinese Academy of Science, Beijing, 100083 People’s Republic of China; Department of Electronic and Electrical Engineering, University College London, Torrington Place, London, WC1E 7JE UK

**Keywords:** Focused ion beam, Nanofabrication, Self-assembly, Droplet epitaxy

## Abstract

Ordered nanodroplet arrays and aligned nanodroplet chains are fabricated using ion-beam-directed self-organization. The morphological evolution of nanodroplets formed on GaAs (100) substrates under ion beam bombardment is characterized by scanning electron microscopy and atomic force microscopy. Ordered Ga nanodroplets are self-assembled under ion beam bombardment at off-normal incidence angles. The uniformity, size, and density of Ga nanodroplets can be tuned by the incident angles of ion beam. The ion beam current also plays a critical role in the self-ordering of Ga nanodroplets, and it is found that the droplets exhibit a similar droplet size but higher density and better uniformity with increasing the ion beam current. In addition, more complex arrangements of nanodroplets are achieved via in situ patterning and ion-beam-directed migration of Ga atoms. Particularly, compared to the destructive formation of nanodroplets through direct ion beam bombardment, the controllable assembly of nanodroplets on intact surfaces can be used as templates for fabrication of ordered semiconductor nanostructures by droplet epitaxy.

## Background

Nanodroplets have been intensively investigated to fabricate various III-V nanostructures, including quantum dot molecules, quantum rings, and nanoholes [1–12]. The unique growth protocols of droplet epitaxy have not only enabled fabrication of complex nanostructures but also led to advanced optoelectronic devices [13–19]. However, laterally aligned nanostructures cannot be obtained by using droplet epitaxy. Fabrication of laterally ordered nanodroplets, which can be used as local sources for epitaxy growth, can address such urgent need of ordered nanostructures in this rapidly growing research field of droplet epitaxy. Focused ion beam (FIB) bombardment has been widely used as a surface preparation and nanopatterning technique for fabrication of self-assembled nanostructures such as nanoripples, nanoneedles, nanoholes, and nanodots [20–25]. Ordered nanostructures by ion bombardment are of interest due to their simplicity and ability to be applied to a wide range of materials. For instance, FIB-induced self-assembly of ordered nanostructures has been reported on metals, semiconductors, and insulators [20, 21, 26–32]. In addition, assisted by ion processing, controlled three-dimensional assembly at mesoscopic scale has been demonstrated recently [33].

Particularly, the recent observation of self-organized nanodroplets on III-V semiconductor substrates has shown a feasible method in forming ordered metallic Ga nanodroplet arrays and opened great opportunities for nanofabrication [34, 35]. Despite the great potential of the ordered Ga nanodroplets in nanomaterial growth, only a few studies have been reported on the evolution and dynamics of nanodroplet self-assembly upon ion beam bombardment. In this paper, the morphological evolution of self-assembled Ga nanodroplets is investigated systematically on the GaAs surface under the influence of focused Ga^+^ ion beam bombardment with different incident angles and beam currents. The self-assembled Ga nanodroplets exhibit higher uniformity and lateral ordering by increasing the incident angle. In addition to incident angles, the ion beam current also plays a critical role in the surface ordering of nanodroplets. In contrast to previous studies, the increase of ion fluence through higher beam currents resulted a higher density but in a similar size. Moreover, via in situ ion beam patterning, Ga nanodroplets were fabricated on intact surfaces via ion-beam-induced Ga atom migration. Ga nanodroplets arranged in different patterns can be simply engineered by adopting different patterns. Upon exposure to group V molecule beam, such as As2, these ordered nanodroplets can be transferred into various types of nanostructures [36]. The presented results may shed light on the dynamics of nanodroplet self-assembly under high-energy ion beam bombardment and generate the building blocks for ordered nanostructured by droplet epitaxy.

## Methods

**Fig. 1:**
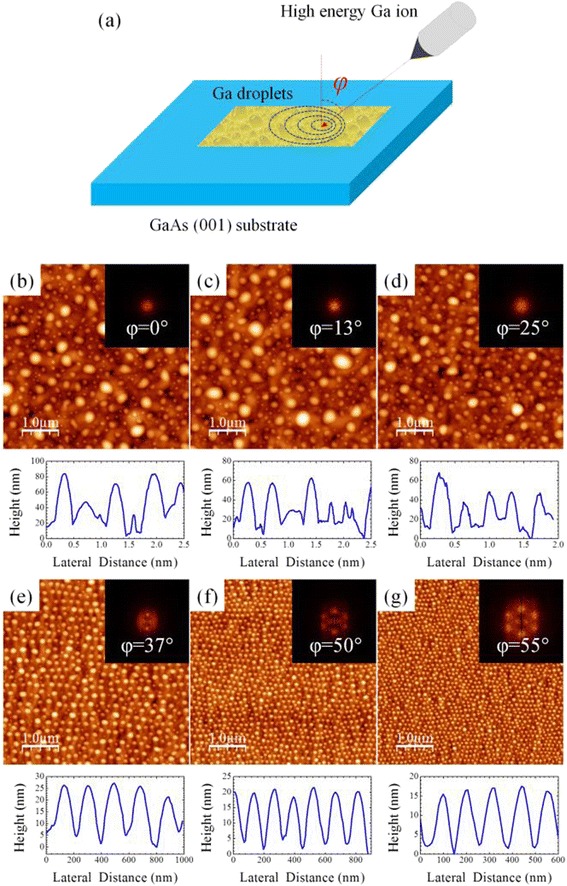
**a** Illustration of the experiment setup. The AFM images, 2D-FFT spectra, and AFM line profiles of Ga nanodroplets formed on the GaAs surfaces with six different ion beam incident angles: **b** 0°, **c** 13°, **d** 25°, **e** 37°, **f** 50°, and **g** 55°. The ion beam current is 0.93 nA, and sputtering time is 5 min

Epi-ready semi-insulating GaAs (001) substrates were used in all experiments. The ion bombardment on the substrates were carried out using a FIB instrument (Hitachi FB-2100, Ga^+^ ion) with a beam-limiting aperture of 80, 150, 300, and 650 μm, corresponding to a beam current of 0.06, 0.26, 0.93, and 3.71 nA, respectively. An acceleration voltage of 10 keV was used for the Ga^+^ ions. Different ion beam incident angles *φ* ranging from 0° to 55° (0°, 13°, 37°, 50°, and 55°) and beam currents from 60 pA to 3.71 nA (0.06, 0.27, 0.93, and 3.71 nA) were used to investigate the evolution of nanodroplets. Figure 1a illustrates the experiment setup. The GaAs surface was bombarded uniformly within an area of 25 × 25 μm in the experiments. Dwell time of 1 μs and sputter time of 5 min were kept constant. For pre-patterned experiments, nanosized ridges were fabricated by electron-beam lithography (EBL) and inductively coupled plasma (ICP) etching. In addition, in situ ion beam milling was also used to form patterns for fabrication of arrays of nanodroplets. During ion beam bombardment, the high vacuum chamber was maintained at a base pressure of 1 × 10^−7^ Torr. After the FIB treatment, the sample surfaces were characterized by scanning electron microscopy (SEM). Atomic force microscopy (AFM) was used to investigate the surface morphologies of nanodroplets formed by ion bombardment. The droplet morphological results of nanodroplets and the two-dimensional (2D)-fast Fourier transformation (FFT) spectrum were analyzed by the software WSxM NanoTec.

## Results and Discussion

Figure 1b–g shows the AFM images of Ga droplets on the GaAs surface after the 10-keV Ga^+^ bombardment beam for 5 min. The ion beam current was 0.93 nA, and the incident angle was varied from 0° to 55°. After critical fluence of ion bombardment, Ga droplets are formed on the GaAs surfaces. The formation of nanosized Ga droplets under ion beam bombardment is caused by the desorption of As after breaking the Ga-As bonds as well as Ga deposition from the Ga^+^ ions. Therefore, the mechanism of self-organized Ga droplets can be understood by the FIB sputtering and surface mass transport. First, during high-energy ion bombardment, the GaAs bonds are broken and As atoms are desorbed from the substrate. Although the high-energy ion beam also sputters off Ga atoms from the GaAs substrate, the preferential sputtering of As atoms leaves a Ga-rich surface. The surplus Ga adatoms then start to diffuse and nucleate into nanosized droplets. At the same time, Ga atoms are supplied and deposited to the surface from the ion beam, which also may contribute to the formation of Ga droplets. For example, at the bombardment condition of a beam current of 0.9 nA at an incident angle of 50°, the fluence of Ga^+^ gains from ion implantation and sputtering is 1.7 × 10^17^ cm^−2^ within a 5-min bombardment time. Assuming unity sticking coefficient, the equivalent thickness of material gain from ion beam deposition is in the order of tens of nanometers. At low ion beam incident angles, Ga droplets exhibit random distribution as shown in Fig. 1b–d. On the other hand, short-range lateral ordering of Ga nanodroplets is observed when the ion beam bombardment is performed at higher angles as shown in Fig. 1e–g.

In order to gain further insight of the ordering processes, the 2D-FFT spectra of the Ga nanodroplets are extracted from the AFM images. The insets in Fig. 1b–g show that the 2D-FFT spectra of the Ga nanodroplet samples are obtained with ion bombardment at different incident angles. Again, the 2D-FFT spectra confirm the self-ordering of nanodroplets on the GaAs surface under ion beam bombardment at high incident angles. When ion beam incident angle is 37°, the 2D-FFT spectrum of the nanodroplets starts to show one-dimensional ordering. When the incident angle increases to 50° and 55°, hexagonal patterns are observed in the 2D-FFT spectra, indicating that the formation of nanodroplets aligned in hexagonal lattices. The AFM images also reveal the morphology evolution of nanodroplets with different incident angles, as shown in Fig. 1. At low small incident angles, the droplet sizes are randomly distributed and big dots are presented on the surface. Increasing incident angles, the droplets become smaller and shorter. When the nanodroplets start to order, the size uniformity of the nanodroplets is also improved. The AFM line profiles in Fig. 1 show that the nanodroplets dramatically shrink their sizes while the periodicity is improved. An anisotropic supply and loss of Ga atoms can be used to explain the formation of self-aligned Ga nanodroplet arrays and nanodroplet evolution on the GaAs surface under Ga^+^ ion beam bombardment at different incident angles [35]. The anisotropic supply and loss of Ga atoms suggest that the profile of energy distribution on the GaAs surface during ion beam bombardment affects nanodroplet ordering. At normal incidence, the distribution of energy on the surface is isotropic and exhibits a circular shape. As a result, the surface diffusion of Ga atoms is random, and Ostwald ripening drives small droplets to merge into bigger and more energetically favorable ones because of their lower surface-to-volume ratio. Therefore, the droplets form randomly with broad size distribution. On the other hand, at off-normal incidence, the energy distribution profile has an anisotropic ellipse contour resulting in directional atom migration on the surface. A consequence of combining the directional atom gain and loss with shadowing and exclusion zone effects is the net supply of Ga atoms between droplets guided by the driving force of the ion beam along the projected beam direction. As a result, the incoming Ga ions can drive the droplets to adjust their location and lead to formation of ordered arrays with sufficient time of ion bombardment. The balance between the Ga gain of droplet from capturing Ga atoms produced at the substrate and the Ga loss resulting from sputtering is attributed to the incident angle-dependent evolution of nanodroplet size.

**Fig. 2:**
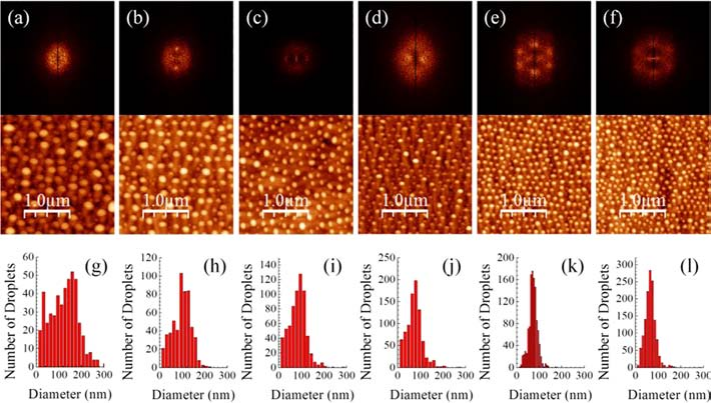
The AFM images and 2D-FFT spectra of Ga nanodroplets formed on the GaAs surfaces with ion beam parameters: **a** ion beam current = 0.27 nA and *φ* = 37°, **b** ion beam current = 0.93 nA and *φ* = 37°, **c** ion beam current = 3.71 nA and *φ* = 37°, **d** ion beam current = 0.27 nA and *φ* = 50°, **e** ion beam current = 0.93 nA and *φ* = 50°, and **f** ion beam current = 3.71 nA and *φ* = 50°. **g**–**l** The nanodroplet size distribution histograms of the AFM images in **a**–**f**, respectively

However, the formation of a hexagonal pattern has not been well explained by the anisotropic supply and the loss of Ga atoms alone. To this end, the effects of ion beam current on the self-organization of Ga nanodroplets have been investigated at incident angles of 37° and 50° to obtain further insight in the ordering patterns of Ga nanodroplets. The ion bombardment was carried out using four different beam currents (0.06, 0.27, 0.93, and 3.75 nA). The AFM images and droplet size histograms are shown in Fig. 2. At a beam current as low as 0.06 nA, only scarce Ga nanodroplets are formed on the surface. However, once the beam current reaches a threshold value, surface ordering of nanodroplets is observed. At an incident angle of 37°, the nanodroplets start to align when using a beam current of 0.93 nA. At a higher incident angle of 50°, surface ordering of nanodroplets is observed at a lower beam current of 0.27 nA. Increasing to even higher beam currents, the surface ordering evolves from one-dimensional chains to two-dimensional hexagonal arrays. For both incident angles, a higher beam current leads to a better surface ordering and size uniformity, as shown in AFM images and histograms of Fig. 2. These results indicate that besides incident angles, the ion beam current (or fluence) also plays a significant role in the in-plane ordering of nanodroplets. Apparently, the ion fluence does not have a significant impact on the energy distribution profiles of ion beams which is mainly dependent on the incident angle. Therefore, the directional mass loss and gain is not the only reason for formation of ordered nanodroplets. Here, we attribute the self-organization of nanodroplets during ion beam bombardment to the shadowing effect and the directional bombardment of Ga ions. New Ga droplets can only nucleate between two adjacent nanodroplets if the pre-existing nanodroplets are displaced far enough; when two adjacent nanodroplets are separated within the diffusion length of Ga adatoms, the deposited Ga will be absorbed by the pre-existing nanodroplets instead of forming new ones. If two adjacent droplets are closely placed, the new nanodroplets can only be generated in a different row but between the two adjacent droplets due to shadowing effects. Therefore, similar to strain-induced self-organization of quantum dots, the nanodroplet nucleation become more uniformly spaced as the Ga ion fluence increases [37]. The directional supply of Ga ions and shadowing effects lead to “self-adjustment” of nanodroplets with characteristic space determined by the ion beam parameters. For the same reasons, the Ga droplets aligned with an adjacent row cannot gain as many Ga atoms as the ones aligned to the gaps of nanodroplets in the adjacent row. On the other hand, because the sputter rates of Ga and GaAs are different, the pre-existing Ga nanodroplets shrink their sizes as the ion fluence increases if Ga gain is restrained by shadowing effects [38]. Therefore, the nanodroplet nucleation positions of adjacent rows are correlated, which results in hexagonal patterns of nanodroplets.

**Fig. 3:**
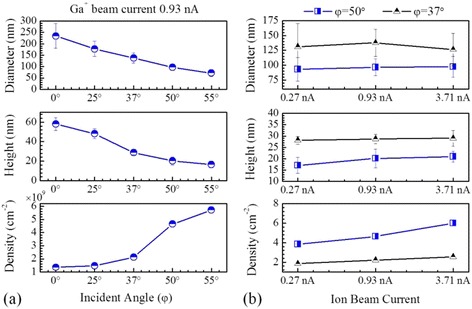
The nanodroplet diameter, height, and density as a function of ion beam **a** incident angle and **b** currents. The nanodroplets were formed with an ion beam current 0.93 nA in **a** and incident angles 37° and 50° in **b**

Figure 3 summarizes morphological parameters of nanodroplets obtained at different ion beam incident angles and currents. The nanodroplets increase density and decrease their dimension as increasing the incident angle. However, at higher beam currents with the same incident angle, the droplets exhibit similar dimensions (slightly decrease in size) but higher density. At a high incident angle, the sputtering of Ga exceeds the absorption of Ga atoms, resulting in negative growth of nanodroplets and an increase in density. Interestingly, a higher ion beam current also leads to more nanodroplets with a similar size instead of creating larger droplets and keeping similar density. Contrary to previous report, which demonstrated that the droplet size increased with increasing ion fluence by extending increasing bombardment period [34], the increase of ion fluence by using a higher beam current does not change the droplet size significantly. It is worth noting that the surface evolution of nanodroplet self-ordering is captured without any surface patterns. After reaching the balance between Ga absorption and sputtering, formation of new nanodroplets is preferred in between pre-existing droplets. New nanodroplets are continually created until the surface is saturated. At the same time, the critical size of nanodroplet is gradually changing due to the change of the balance between the sputtering of Ga from the nanodroplets and Ga absorption from the surface after formation of new nanodroplets as evidenced in Fig. 3.

**Fig. 4:**
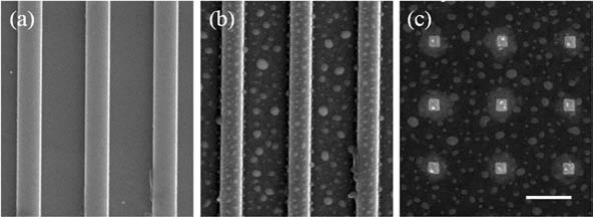
**a** Nanosized GaAs strips fabricated by EBL and ICP etching. Localized nanodroplets forming on **b** GaAs nanostrips and **c** GaAs nanomesas under 0.93 nA normal incident ion beam bombardment for 1 min. The *scale bars* are 1 μm

While the surface arrangement, dimension, and density of nanodroplets can be well controlled by adjusting the ion beam parameters as shown in Figs. 1, 2, and 3, site-controlled self-assembly of nanostructures are preferred in many applications, such as quantum information processing. One way to form site-controlled formation of nanodroplets is to pattern the substrate prior to ion bombardment. Figure 4a shows ridges fabricated by electron-beam lithography (EBL) and inductively coupled plasma (ICP) etching. After ion beam bombardment, chains of nanodroplets are formed on the ridges in Fig. 4b. Due to the confinement of the ridges, the nanodroplets on the ridges show improved size uniformity and surface ordering. The reduced size of nanodroplets is also attributed to the limited Ga diffusion on the pre-patterned ridges. The pre-patterned template can also lead to form nanodroplets with greater complexity. Figure 4c shows nanodroplets can be created and confined in localized areas through pre-patterning the substrates with squares in sub-microscale. In Fig. 4b, c, the nanodroplets can be located in the patterned area but the ordering is rather poor. For example, as shown in Fig. 4c, either single or double nanodroplets can be found in the pre-patterned nanosized squares. As demonstrated in Figs. 1, 2, and 3, the surface ordering of nanodroplets can be improved via optimized ion beam parameters, which, however, inevitably require extended ion bombardment. The increased ion dose can destroy the template, and site-controlled fabrication of nanodroplets cannot be obtained. Moreover, the ion bombardment induces radiation damages in the crystal [39].

**Fig. 5:**
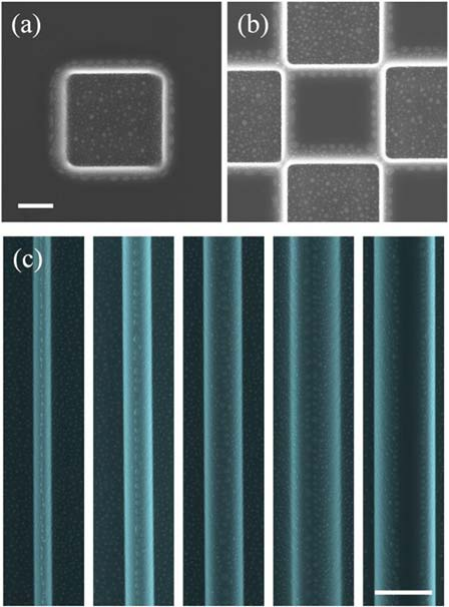
**a** The SEM image of Ga nanodroplets form around a square area irradiated by ion beam. **b** The SEM image of directed assembly of Ga nanodroplets via in situ ion beam patterning. **c** SEM images of nanodroplet chains forming on ridges of 0.4, 0.8, 1.2, 1.6, and 2.0 μm. The incident angle is normal to the sample surface in **a** and **b** and 35° in **c**. The bombardment time is 1 min. The *scale bars* are 2 μm

It is highly desirable to form nanodroplets on surfaces without crystal damage induced by high-energy ions. To form ordered Ga nanodroplets in intact GaAs surfaces can be achieved by ion-beam-induced Ga migration [40, 41]. Random motion of Ga nanodroplets can be induced by ion beam bombardment due to ion-beam-induced anisotropic mass transport and thermal fluctuation [41–43]. Figure 5a shows the formation of Ga nanodroplets around a square area bombarded with 0.93-nA Ga^+^ ion beam for 1 min. Ga atoms migrate to the edge of the irradiated area and then nucleate into nanodroplets during ion beam bombardment. As a result, nanodroplets can self-organize during ion beam patterning of the surface. Similar to pre-patterning, in situ ion beam milling can also lead to form nanodroplets arrays with greater complexity. As shown in Fig. 5b, nanodroplets on the intact GaAs surface can be directly formed by the ion beam bombardment patterns. Under ion beam bombardment, a Ga-rich area is created. The migration of Ga atoms from the Ga-rich area to the non-bombarded area makes the boundary of the ion beam patterning area to be the preferred nucleation sites for nanodroplets. As demonstrated in Fig. 1, normal incidence of ion beam tends to form random distribution of nanodroplets. In order to obtain improved uniformity of nanodroplets, off-normal incidence of ion beam is required. Figure 5c shows that aligned nanodroplets have been created during in situ patterning of GaAs trenches using ion beam bombardment at off-normal incidence. The ion beam incident angle, current, and duration are 35°, 0.93 nA, and 1 min, respectively. Compared to normal incident bombardment shown in Fig. 5a, b, the size distribution in Fig. 5c is clearly improved. The ion milling of substrates into trenches also leads to formation of nanodroplet chains on the ridges. By controlling the separation of the trenches (or ridge width), the surface arrangement of the nanodroplets chains can be manipulated. For narrow ridges (0.4 and 0.8 μm), nanodroplets are aligned into one row. With increasing ridge width, the one row of nanodroplets starts to break into two rows as shown in Fig. 5c: 1.2 and 1.6 μm. Further increase in the ridge width results in clearly separated two rows of nanodroplets aligned along the edge of the ridges as the ridge width becomes larger than the diffusion length of Ga atoms. This study shows that the self-organization of nanodroplets can also be well controlled by controlling the ion milling patterns.

## Conclusions

Ordering of Ga droplets occurs through self-assembly on the GaAs substrate under Ga ion bombardment at off-normal incidence angles. The morphological evolution of the nanodroplets is systematically investigated under various ion beam bombardment conditions. The well-ordered Ga droplets can be obtained with off-normal incidence of high-energy ion beams. The surface arrangement, size, and density of the nanodroplets can be finely tuned by adjusting the ion beam parameters. With increase of incident angle, the Ga droplets exhibit smaller size distribution and smaller height but higher density. With increase in ion fluence by increasing ion beam current, lateral ordering can be also improved with a similar size but higher density. The formation of self-ordered Ga nanodroplets on undamaged GaAs surface has also been achieved by taking advantages of the ion-beam-induced surface migration of Ga atoms. The ordering of Ga nanodroplets can be directed by controlling the ion milling patterns. The method of fabrication of ordered nanodroplets presented in this study provides another essential step towards fabrication of advanced semiconductor nanostructures by using droplet epitaxy.
